# Creating ‘obesogenic realities’; do our methodological choices make a difference when measuring the food environment?

**DOI:** 10.1186/1476-072X-12-33

**Published:** 2013-07-02

**Authors:** Thomas Burgoine, Seraphim Alvanides, Amelia A Lake

**Affiliations:** 1 UKCRC Centre for Diet and Activity Research (CEDAR), Box 296, Institute of Public Health, Forvie Site, Robinson Way, University of Cambridge, Cambridge, CB2 0SR, UK; 2 Geography and the Built Environment, Ellison Building, Northumbria University, Newcastle upon Tyne, NE1 8ST, UK; 3 Centre for Public Policy and Health, School of Medicine, Pharmacy and Health, Wolfson Research Institute, Queen’s Campus, Thornaby, Durham University, Stockton-on-Tees, TS17 6BH, UK

**Keywords:** Obesogenic Environments, Foodscape, Methods, Exposure, Density, Proximity

## Abstract

**Background:**

The use of Geographical Information Systems (GIS) to objectively measure ‘obesogenic’ food environment (foodscape) exposure has become common-place. This increase in usage has coincided with the development of a methodologically heterogeneous evidence-base, with subsequent perceived difficulties for inter-study comparability. However, when used together in previous work, different types of food environment metric have often demonstrated some degree of covariance. Differences and similarities *between* density and proximity metrics, and *within* methodologically different conceptions of density and proximity metrics need to be better understood.

**Methods:**

Frequently used measures of food access were calculated for North East England, UK. Using food outlet data from local councils, densities of food outlets per 1000 population and per km^2^ were calculated for small administrative areas. Densities (counts) were also calculated based on population-weighted centroids of administrative areas buffered at 400/800/1000m street network and Euclidean distances. Proximity (street network and Euclidean distances) from these centroids to the nearest food outlet were also calculated. Metrics were compared using Spearman’s rank correlations.

**Results:**

Measures of foodscape density and proximity were highly correlated. Densities per km^2^ and per 1000 population were highly correlated (r_s_ = 0.831). Euclidean and street network based measures of proximity (r_s_ = 0.865) and density (r_s_ = 0.667-0.764, depending on neighbourhood size) were also highly correlated. Density metrics based on administrative areas and buffered centroids of administrative areas were less strongly correlated (r_s_ = 0.299-0.658).

**Conclusions:**

Density and proximity metrics were largely comparable, with some exceptions. Whilst results suggested a substantial degree of comparability across existing studies, future comparability could be ensured by moving towards a more standardised set of environmental metrics, where appropriate, lessening the potential pitfalls of methodological variation between studies. The researchers’ role in creating their own obesogenic ‘reality’ should be better understood and acknowledged.

## Introduction

With the scale of the obesity epidemic ever increasing, there has been a recent and growing body of literature that suggests an environmental contribution of the food environment (foodscape) to dietary choices and obesity [1]. Food choices are made in context, within our respective foodscapes at micro and macro levels [2,3], and hold the potential to shape our behaviour. Research studies of the food environment at the neighbourhood level have reported a variety of outcomes and have employed a range of methodologies [4]; many use Geographical Information Systems (GIS) to objectively measure the potential environmental influence on individuals’ behaviours and overall health.

Practically, there has been increased attention paid to the ways in which environmental foodscape attributes pertaining to weight can be modelled using GIS [5]. Many studies of the ‘obesogenic’ [1] food environment utilise measures of density and proximity to measure food ‘access’. Food outlet density is a measure designed to reflect the range or ‘intensity’ of any given food outlet type, in terms of the number of food outlets present around an individual [6,7]. Food outlet proximity is usually the distance to the nearest food outlet, with distance inversely related to utilisation [8,9]. Charreire *et al.*[4] systematically demonstrated the pervasiveness of density and proximity metrics in the published literature. However, the substantive differences between density and proximity metrics, beyond their theoretical distinction, are under-studied. Do areas with high food outlet density always offer residents food outlets at a closer proximity? Could ensuing analyses be simplified by using only one foodscape exposure metric? Two previous studies have concluded that both measures “tell a consistent story about food access” [9,10], questioning the extent to which both measures are necessary, considering the effort required to compute multiple metrics and the problems associated with covariance in later analyses; more research is required to better understand this issue.

Moreover, precise definitions of density and proximity vary between studies. For example, when evaluating proximity, studies have used straight line (Euclidean) distance [11-13], or street network distance to the nearest food outlet [14-24], which in reality may be very different distances. Despite this, only one US study has addressed differences in food outlet proximity estimates when using either street network or Euclidean approaches [9]; no international comparison has been provided in the literature to date. Other common approaches to estimating food outlet access include: studies using food outlet density per head of population [6,10,16,25-37], whilst others, density per square unit of area [26,28,38]; all of these studies have calculated exposure within some previously defined administrative boundary (census tracts, electoral wards and so on), whereas others have chosen to use GIS to buffer study participants [39-49], or where these locations are unknown, the centroids of these administrative areas (geographic- or population-weighted) [11], creating ‘neighbourhoods’ at a range of spatial scales (for example 400/800/1000), which here we proprietarily refer to as ‘pseudo-individual’ neighbourhoods; using the latter approach, or again the exact locations of study participants, both Euclidean [11,39-45], and street network based definitions of neighbourhood have been employed [49,50]. Table 1 gives more detail about these common approaches to estimating food outlet access, but a recent methodological review *comprehensively* described the wide range of approaches to calculating density and proximity taken in the literature [4], including others, such as novel inverse distance weighting (IDW) approaches to calculating food access [48], that have been used less frequently in the literature to date. This methodological heterogeneity is potentially problematic, yet the extent to which it may contribute to a lack of comparability between foodscape research findings is unknown. A methodologically heterogeneous evidence-base is often simply the bi-product of development within a science over time, and may not be associated with grave implications for inter-study comparability. However, it is nonetheless important to continue to work to understand the possible consequences of this evolution. Recent studies have begun to employ multiple metrics of foodscape exposure, and have considered the implications of methodological nuance between types of density metrics and between types of proximity metrics [9,39,40,51]. However direct comparisons of metrics were either not made or the range of metrics assessed was limited. In one study [9], the two density metrics compared represent only two of the many methodological options available for objectively capturing food outlet exposure. A more comprehensive comparison of types of density and proximity metrics is required in order to understand what implications our methodological choices have in determining estimates of geographic access to food outlets.

**Table 1:** Food environment exposure metrics compared in this study, and precedent for their use in the literature

**Variable**	**Description**	**Precedent for use**^**a**^
**LSOA**		
*Density per: *		
1000 population	Density of food outlets per 1000 population/per km^2^, per LSOA	O’Dwyer & Coveney [25] | Maddock [26] | Mehta & Chang [27] Ball *et al.*[6] | Moore & Diez Roux [28] | Chou *et al.*[29] Mobley *et al.*[30] | Simmons *et al.*[31] Sturm & Datar [32] Black *et al.*[33] | Powell *et al.*[34] | Burgoine *et al.*[10] Cummins *et al.*[35] | Reidpath *et al.*[36] | Macdonald *et al.*[37] Macdonald *et al.*[16]
km^2^		Block *et al.*[38] | Maddock [26] | Moore and Diez Roux [28]
**LSOA centroid**		
*Density per buffer at:*		
400 m Euclidean radius 800 m Euclidean radius	Counts of food outlets within 400/800/1000 m Euclidean radius buffers from population-weighted centroids of LSOA	Austin *et al.*^c^[39] | Currie *et al.*^c^[40] Austin *et al.*^c^[39] | Jeffery *et al.*^c^[41] | Laraia *et al.*^c^[42] Timperio *et al.*^c^[43] | Currie *et al.*^c^[40] Spence *et al.*^c^[44]
1000 m Euclidean radius		Apparicio *et al.*[11] | Smoyer-Tomic *et al.*[45] | Seliske *et al.*^*c*^[46]
400 m Street Network 800 m Street Network 1000 m Street Network	Counts of food outlets within 400/800/1000 m street network buffers from population-weighted centroids of LSOA	Smith *et al.*^*c*^[47] Smoyer-Tomic *et al.*[50] | Harrison *et al.*[48] Larsen & Gilliland^c^[49] | Seliske *et al.*[46]
*Proximity using:*		
Euclidean distance Street network distance	Euclidean or street network distance (m) from LSOA population-weighted centroids to nearest food outlet	Apparicio *et al.*[11] | Winkler *et al.*[12] | Bodor *et al.*[13] Zenk *et al.*^b^[14] | Pearce *et al.*[15] | Macdonald *et al.*[16] | Smith *et al.*[17] Pearce *et al.*[18] | Sharkey *et al.*[19] | Pearce *et al.*^d^[20] Sharkey & Horel [21] | Block *et al.*^*c*^[24] | Burdette & Whitaker [22] | Frank *et al.*^*c*^[23]

^a^Methodologically similar, not necessarily using LSOAs; using imperial or metric measurements; geographic- or population-weighted centroids.

^b^Manhattan block distance used as an alternative to street network distance.

^c^Buffer sizes employed around known home or school address.

^d^Used travel time rather than travel distance.

Our study used GIS to compute a range of commonly employed foodscape density and proximity metrics in the North East of England, UK. The research had two principle aims. Firstly, to compare food outlet density and proximity metrics to one another using correlation analysis. Secondly, to compare different metrics of density and proximity in a sensitivity analysis, to determine whether methodological differences in the calculation of each type of metric might serve to limit comparability between existing research findings. Comparisons made were as follows: 1) administrative area density per 1000 population vs. administrative area density per km^2^; 2) administrative area densities vs. 400/800/1000 m ‘pseudo-individual’ densities; 3) 400/800/1000 m ‘pseudo-individual’ densities, when using either Euclidean vs. street network buffers; 4) Euclidean vs. street network measures of proximity from population-weighted LSOA centroids. These four binaries of methodological comparison represent common-sense comparisons, and have been suggested as necessary in empirical work and systematic reviews in the literature [4,11,51-53].

## Methods

The study area for this research was the former North East Government Office Region, UK, an environmentally heterogeneous area covering 8676 km^2^. Locations of food outlets (n = 14,454) were sourced from local councils (n = 23) under Freedom of Information (FOI) requests (for more details on the FOI Act 2000, see http://www.legislation.gov.uk/ukpga/2000/36/data.pdf). This data represents the most accurate secondary source of food outlet location data in the UK [54,55]. Food outlets were categorised into ‘Food Bought-’ (n = 3793) and ‘Food Consumed Out of the Home’ (n = 10,661) types, based on the likely site of food preparation [56]; see Burgoine [57] for full details. For brevity, only the geographies of ‘Food Bought Out of the Home’ outlets were considered in this study (‘supermarkets’, ‘convenience’, ‘discount’ and ‘department’ stores, plus ‘specialist’ retailers (butchers, delicatessens, bakers, fishmongers, confectioners, greengrocers, health, organic, fair trade, artisan, sweet and oriental stores)); results not presented showed no differences in findings when analysing ‘Food Consumed Out of the Home’ outlets (‘restaurants’, ‘pubs’, pizzerias’, ‘fast food’, ‘cafés’/coffee shops’, ‘takeaways’, ‘sandwich shops’, ‘bakers – retail’, ‘hotels’, ‘entertainment’, ‘health and leisure’, ‘novelty stores’ and ‘pharmacies’). Using lookup tables from GeoConvert (http://geoconvert.mimas.ac.uk/), food outlet locations were mapped based on their postcodes using ArcGIS 9.3 (ESRI Inc., Redlands, CA). Recent precedent has been set in the literature for geocoding at the postcode level in the UK [47].

Details of the exposure metrics deduced are shown in Table 1 (the ‘Variable’ column). Neighbourhood densities of food outlets were calculated per 1000 population (population data from the 2001 UK census) and per km^2^ within Lower Super Output Areas (LSOAs); these are small administrative areas (median size 0.49 km^2^), each containing roughly 1500 residents (available from UKBORDERS, http://edina.ac.uk/census/). Densities (counts of food outlets) were also calculated within 400m, 800m and 1000m Euclidean and street network buffers from the population-weighted centroids of LSOAs. We refer to these buffered LSOA centroids as ‘pseudo-individual’ neighbourhoods on the assumption that food access at this calculated location is representative of the rest of the LSOA. Street network data were provided by Ordnance Survey as part of their MasterMap Integrated Transport Network (ITN). Proximity, Euclidean and street network distance to the nearest food outlet, was also calculated from these population-weighted centroids. Precedent for the frequent use of each metric, is also shown in Table 1, informed by recent systematic review articles [4,52,53,58], which should be referred to for a *comprehensive* review of the literature. The metrics compared and contrasted in this study represent those that have been widely employed in obesogenic environment studies to date.

### Statistical analysis

Analyses were performed throughout using SPSS 17.0 (SPSS Inc., Chicago, 2006). Descriptive statistics (medians, interquartile ranges, and ranges) for our environmental metrics are presented first. As our exposure metrics were continuous variables, differences *between* density and proximity metrics, and *within* types of density and types of proximity metrics were assessed using correlation analyses. We used Spearman’s rank correlations because the vast majority of our access metrics were not normally distributed, with their distributions tending to be positively skewed. When comparing density and proximity metrics directly, we present the inverse of the correlation co-efficient obtained. As higher proximity values actually relate to greater distances (worse access to the food outlet), this allows, for example, a positive co-efficient to be interpreted as greater density allied with greater proximity (less distance).

## Results

**Figure 1:**
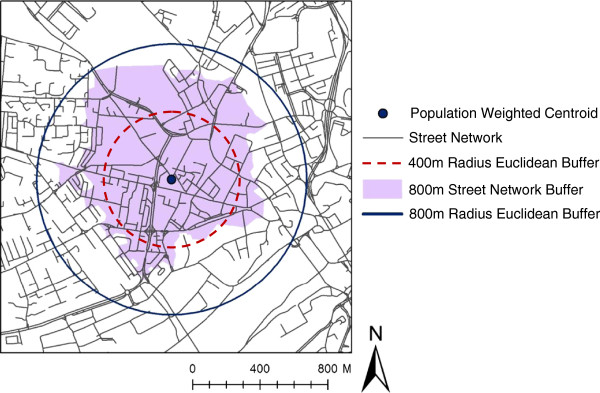
**Comparing 800m/400m Euclidean buffers to 800m street network buffers.** © Crown Copyright/database right 2012. An Ordnance Survey/EDINA supplied service.

Descriptive statistics for the exposure metrics created are shown in Table 2. The median number of food outlets per 1000 population was 0.8, whilst the median number per km^2^ was 2.0. There was a linear increase in median access as neighbourhood buffer sizes increased, as expected, and more so for Euclidean than street network buffers (which tend to be smaller, as illustrated in Figure 1). Median Euclidean distance to the nearest food bought out of the home outlet was 239.8 m, but 398.8 m when constrained to the street network, and with a much greater range.

**Table 2:** Descriptive statistics for exposure metrics across administrative areas (n = 1656)

		**Median**	**Interquartile range**	**Minimum**	**Maximum**
**Density**	Per 1000 population	0.8	0.0-2.0	0.0	35.0
	Per km^2^	2.0	0.0-6.2	0.0	105.4
	Per 400m Euclidean buffer	2.0	1.0-4.0	0.0	40.0
	Per 800m Euclidean buffer	7.0	3.0-12.0	0.0	81.0
	Per 1000m Euclidean buffer	10.0	5.0-18.0	0.0	94.0
	Per 400m street network buffer	0.0	0.0-2.0	0.0	34.0
	Per 800m street network buffer	3.0	1.0-6.0	0.0	63.0
	Per 1000m street network buffer	4.0	2.0-9.0	0.0	80.0
**Proximity**	Euclidean distance	239.8	141.6-379.7	6.1	8535.4
	Street network distance	398.8	229.8-652.2	0.2	17886.4

### Density compared to proximity

This section compares food outlet density and proximity measures throughout the study area. Table 3 shows Spearman’s rank correlations between density and proximity exposure metrics (all p < 0.001). Two salient trends emerged: 1) relationships *within* categories of density/proximity were moderately to strongly correlated in uniform directions (co-efficients ranging between 0.299 and 0.926 for density metrics (mean r_s_ = 0.615); 0.865 for the relationship between proximity metrics); 2) relationships *between* categories of density/proximity were also similar, with co-efficients having positive signs (i.e. all measures of density are moderate to strongly positively correlated with measures of proximity, and vice versa).

**Table 3:** Spearman’s rank correlations between LSOA (n = 1656) density and proximity measures*

		**Density**								**Proximity**	
		**(1)**	**(2)**	**(3)**	**(4)**	**(5)**	**(6)**	**(7)**	**(8)**	**(9)**	**(10)**
**Density**	Per 1000 population (1)	1.000	0.831	0.549	0.354	0.299	0.494	0.474	0.434	0.512^a^	0.478 ^a^
	Per km^2^ (2)		1.000	0.658	0.534	0.512	0.575	0.584	0.570	0.640 ^a^	0.579 ^a^
	Per 400m Euclidean buffer (3)			1.000	0.653	0.586	0.667	0.764	0.718	0.720 ^a^	0.674 ^a^
	Per 800m Euclidean buffer (4)				1.000	0.926	0.445	0.769	0.854	0.474 ^a^	0.474 ^a^
	Per 1000m Euclidean buffer (5)					1.000	0.411	0.695	0.783	0.442 ^a^	0.442 ^a^
	Per 400m street network buffer (6)						1.000	0.610	0.550	0.751 ^a^	0.844 ^a^
	Per 800m street network buffer (7)							1.000	0.918	0.589 ^a^	0.657 ^a^
	Per 1000m street network buffer (8)								1.000	0.543 ^a^	0.593 ^a^
**Proximity**	Euclidean distance (9)									1.000	0.865
	Street network distance (10)										1.000

* All results p < 0.001.

^a^ Inverse of correlation co-efficients presented to aid interpretation between, for example, greater density (higher exposure) and greater proximity (lower exposure).

### Heterogeneity within exposure metrics

This section examines the extent to which methodological heterogeneity *within* exposure metrics may impact upon comparability between findings in the literature to date. We make four necessary comparisons, as outlined in the aims.

#### Density per 1000 population vs. density per km^2^

Correlation analysis (Table 3) showed that whether LSOA density of ‘Food Bought-’ outlets is calculated per 1000 population or per km^2^, both measures were correlated very strongly (r_s_ = 0.831, p < 0.001) giving similar impressions of food access throughout the study area.

#### Area level vs. 400/800/1000m ‘pseudo-individual’ level density

Table 3 showed that only moderate/moderately-strong correlations existed between area level and ‘pseudo-individual’ level metrics, ranging between r_s_ = 0.299 and r_s_ = 0.658. Whilst they are all significant (p < 0.001) and in the same positive direction, these correlations suggest that we could be less sure of comparing results across studies that utilise these different approaches. The specific degree to which this statement holds true was attenuated by the metric examined: area and ‘pseudo-individual’ metrics were more comparable at smaller Euclidean and street network buffer distances, largely decreasing in strength at 800m, and further still at 1000m. The more nuanced street network buffers of ‘pseudo-individual’ density were largely more comparable with area level metrics, however, the 400m Euclidean buffer was the most similar (r_s_ = 0.549 for LSOA density per 1000 population, r_s_ = 0.658 for LSOA density per km^2^, both p < 0.001).

#### Street network vs. Euclidean 400/800/1000m ‘pseudo-individual’ level density

Correlation results (Table 3) showed that levels of ‘pseudo-individual’ density were similar whether using a Euclidean or street network approach (at the same distance), and increasingly so when accounting for larger neighbourhoods (400m r_s_ = 0.667; 800m r_s_ = 0.769; 1000m r_s_ = 0.783, all p < 0.001). 400m Euclidean neighbourhood densities were however more strongly correlated with 800m street network densities (r_s_ = 0.764), and 800m Euclidean densities with 1000m street network densities (r_s_ = 0.854).

#### Street network vs. Euclidean proximity

Results presented in Table 3 showed that ‘Food Bought-’ proximity was comparable when assessed via Euclidean and street network distances (r_s_ = 0.865, p < 0.001). Much like the similarity displayed between Euclidean and street network ‘pseudo-individual’ neighbourhoods, this finding suggested that there was a substantial degree of comparability between the two metrics.

## Discussion

This paper contributes to obesogenic foodscape research in two ways. Firstly, measures of density and proximity were compared to assess the extent to which they represent different facets of the foodscape. These measures are theoretically distinct and commonly differentiated in the literature, and it has been argued that to use just one metric is to do a disservice to the range of ways in which environments may affect behaviour [11]. However, using both metrics may lead to multicollinearity issues in ensuing analyses, resulting from covariance between the two metrics. Further, opting for either of the two metrics may provide a ‘shortcut’ for researchers wishing to employ only a single, representative metric of foodscape exposure. Indeed, correlation analyses presented here provided tentative evidence that these measures represent the foodscape in very similar ways, suggesting that neighbourhoods with high food outlet density were also those where residents live within closer proximity to food, supporting previous findings [9,10]. However, this study does not represent definitive evidence that this is always the case, and opposite results have been observed [11]. Furthermore, although incidental, at the very least each measure may be susceptible to a different level of measurement error [59]. For example, where food outlet location data is not totally comprehensive, measures of food outlet density may be more prone to error (systematic underestimation of exposure) than measures of proximity (which, by chance, may remain completely accurate).

Secondly, this paper evaluated the extent to which methodological heterogeneity in calculating an exposure metric (density or proximity) contributes to a lack of inter-study comparability in findings. To some extent, results here suggested that such criticisms may be unfounded: measures of proximity from population-weighted centroids were highly correlated when using Euclidean or street network distance, corroborating findings from the only one comparable previous US study [9]. In addition, measures of ‘pseudo-individual’ density using Euclidean or street network distances were nearly equal at similar geographical scales, as has also been found elsewhere [51,60], whilst becoming more similar at greater distances. Buffer distances beyond 1000m have been used in the literature [6,11,12,25] and we could therefore expect even greater convergence. This said, there was evidence to suggest that Euclidean buffers were most comparable to marginally larger street network buffers: 400m Euclidean buffers most strongly correlated with 800m street network buffers and 800m Euclidean buffers most strongly correlated with 1000m street network buffers, confirming similar results from Thornton *et al.*[51]. This is likely to be because in general, Euclidean buffers result in a larger footprint, thus encapsulating more food stores, as illustrated in Figure 1. Statistical investigations also reported density per km^2^ and per 1000 population to be similar, inviting comparability across this methodological binary.

Measures of density at the area or ‘pseudo-individual’ level were only moderately correlated with one another, suggesting a limited extent to which findings across this divide should be compared. Unfortunately, this divide across metrics at the area or ‘pseudo-individual’ level separates two large bodies of academic work (Table 1), where studies have either located individuals within administrative area neighbourhoods, or created bespoke neighbourhood buffers around said individuals. Therefore, it may be of benefit to the field if a single (area or ‘pseudo-individual’) density metric were to be used in future research to maximise inter-study comparability, wherever possible. At the very least, studies should consider providing a rationale for their preference of approach to density calculation, whilst better and more fully acknowledging the assumptions and limitations inherent to either choice.

Beyond implying comparability across studies then, results suggest that there may only be small gains made from using street network distances for measures of ‘pseudo-individual’ density or proximity. This may be important when time or resources are at a premium or where street network data is unavailable. However, we disagree with Sparks *et al.*[9] who conclude that this equates to a reduced “computational burden on those wishing to use GIS methodology” – from which we infer that through not having to use more complex street network data, the usability of GIS methods is increased. It is argued here that there is a ‘tipping point’, where the value of using increasingly detailed metrics begins to diminish in relation to the computational effort required to create them. However, as we know little of this ‘tipping point’, and we should be mindful of scale differences (calculating street network availability may be more critical at 400m radii than 1000m radii, for example), we should always try to do the best that we can, even when confronted with technological challenges.

We argue here that we need to further consider how we can advance our methods and our metrics of exposure in objective studies of obesogenic food environments. Some studies have already sought to use measures of variety in relation to the food environment [6,11,32]; for example, the ratio of fast food to full service restaurants [32], designed to complement measures of density and proximity. Others have begun to use inverse distance weighted (IDW) measures of facility access, which to some extent ameliorate concerns arising over the relatively arbitrary definitions of ‘neighbourhood’ applied throughout the literature [48]. In reality however, it may never be possible, or even appropriate, to reach a point where even a well-conceived measure of food access can become a universal standard, suitable for use across all studies, as has been tentatively suggested [61]. Other methodological differences between studies – in statistical techniques, or in terms of study populations and their characteristics, which might vary between countries for example, and so on – ensure that two studies will rarely, if ever, be completely comparable to one another. This does not detract however from the importance of attempting to understand the implications of such diversity, which is what we have begun to address here. A valid and reliable research evidence base, where differences in study findings can be fully understood and appreciated with respect to the methods used, from which conclusions about neighbourhood level effects on diet can be accurately drawn, will be absolutely critical in justifying neighbourhood interventions or pilot interventions designed to promote health, such as restricting the clustering of unhealthy food retailers.

This paper has a number of limitations. In this study we did not compare all foodscape metrics employed in the field to date; instead, we focused on many of the most commonly used metrics in order to relate to as much of the field as possible. Also, we did not investigate the entire foodscape here; different relationships may have been found for ‘Food Consumed Out of the Home’ outlets, although work not presented suggests this is not the case. There is also little compelling logic to suggest that the relationships tested here might be systematically biased according to the type of food outlet selected for study. Furthermore, the category of ‘Food Bought Out of the Home’ includes outlet types such as ‘supermarkets’ and ‘convenience stores’, density and proximity of which are frequently assessed in the literature. We did not have access to data on the exact locations of participants in this study, hence measures of proximity and some measures of density were calculated from administrative area population-weighted centroids, where we assumed at least one individual to live. This is an approach adopted in the literature where participant location data has not been available [11,20], however we acknowledge that this could constitute a type of ‘errors-in-variables’ bias and that exposure throughout any given administrative area will vary [62], and would be likely to decrease away from population-weighted centroids. Our approach of using population-weighted centroids was at least consistent between areas. External validity in findings cannot be assumed, and we cannot rule out that results here may be particular to the North East of England, despite the large and heterogeneous study area, and the similarities between the study area and many other regions of the UK, notably in terms of its diverse socio-economic profile, with which we know foodscape exposure varies [15,35,37]. Lastly, we acknowledge that a greater density or proximity of food does not necessarily equate to more utilisation of these facilities. Considerations such as transport preferences, motivation to walk, economic factors, neighbourhood perceptions and so on will all contribute broadly to ‘access’ beyond purely a geographic perspective. We know for example that in Newcastle upon Tyne, in the North East of England, adults conducting their main supermarket shop on foot travel a median distance one-way of 510 m, as compared to 2528 m for those with access to a car [63]. It is also worth considering that the vast majority of food environment exposure studies have tended to focus exclusively on residential neighbourhood exposure, despite the apparent necessity of accounting for time spent in wider ‘activity spaces’ [64], too. This said, our study compared foodscape metrics that are widely used in the field, rendering this research highly relevant, regardless of whether these previous studies conceived of access in a purely spatial sense or otherwise.

## Conclusions

Our findings should be viewed in the context of creating different ‘obesogenic realities’ during the research design and analysis process. Despite occasional assertions to the objectivity of the “scientific approach”, quantitative researchers are ‘critical agents’ in the GIS process [65,66], and are required throughout their research to make a myriad of choices; the potential for these choices to impact upon results has been suggested elsewhere [67], and demonstrated to some extent here. In this way we create our own obesogenic realities and we should remain reflexive towards, and critical of these creations, in order to improve results and to chart a course for better future research. ‘Researcher bias’ has been well discussed in qualitative literatures, where data is often seen as being ‘generated’ rather than ‘collected’ as a reflection of the investigator’s input into the research process [68,69]. Such issues of bias are less frequently discussed in quantitative discourse, particularly in relation to GIS, despite their apparent poignancy, although feminist critiques of GIS amongst others seek to increasingly understand the “exclusions, silences, and marginalizing [*sic*] power of our representations” [70-72]. Objective GIS studies are often based on the notion of ‘technological determinism’ [65,69], yet the extent to which this is possible is arguably limited. Leszczynski [73] warns against committing the ‘ontic fallacy’, whereby we unquestioningly accept our representation of reality to “mirror nature”, and through failing to acknowledge the impact of the choices we make as researchers on the results we obtain, we risk falling into the ontological trap of what can truly be known. This paper demonstrates the need for such concern by contrasting an extensive range of environmental metrics and demonstrating that at least in some instances, the choices that we make do matter.

Ideally, our understanding of how individuals interact with their food environments should advance alongside available data sources and expertise, to allow similar but appropriate metrics of both density and proximity to be used between studies. This would facilitate the creation of a methodologically consistent evidence base, upon which to ascertain an objective environmental influence upon individuals, whilst not having to make excuses for methodological variation. Whilst desirable however, this may not be possible or indeed appropriate. Nevertheless, this study helps us to understand the current state of evidence in obesogenic environment research, by demonstrating that heterogeneity between most exposure metrics is not necessarily problematic.

## Competing interests

The authors declare they have no competing interests.

## Authors’ contributions

The study design was jointly devised by TB, SA and AL. TB was responsible for data collection, and led on data analysis, in consultation with SA and AL. TB drafted the manuscript. All authors read and approved the final manuscript.
